# Pharmaceutical and non-pharmaceutical interventions for controlling the COVID-19 pandemic

**DOI:** 10.1098/rsos.230621

**Published:** 2023-12-20

**Authors:** Jeta Molla, Suzan Farhang-Sardroodi, Iain R. Moyles, Jane M. Heffernan

**Affiliations:** ^1^ Department of Mathematics and Statistics, York University, Toronto, Ontario, Canada; ^2^ Centre for Disease Modelling (CDM), Mathematics Statistics, York University, Toronto, Ontario, Canada; ^3^ Modelling Infection and Immunity Lab, Mathematics Statistics, York University, Toronto, Ontario, Canada; ^4^ Department of Mathematics, University of Manitoba, Winnipeg, Manitoba, Canada

**Keywords:** susceptible–infectious–recovered model, COVID-19, physical distancing, pharmaceutical (vaccination) and non-pharmaceutical interventions, waning immunity

## Abstract

Disease spread can be affected by pharmaceutical interventions (such as vaccination) and non-pharmaceutical interventions (such as physical distancing, mask-wearing and contact tracing). Understanding the relationship between disease dynamics and human behaviour is a significant factor to controlling infections. In this work, we propose a compartmental epidemiological model for studying how the infection dynamics of COVID-19 evolves for people with different levels of social distancing, natural immunity and vaccine-induced immunity. Our model recreates the transmission dynamics of COVID-19 in Ontario up to December 2021. Our results indicate that people change their behaviour based on the disease dynamics and mitigation measures. Specifically, they adopt more protective behaviour when mandated social distancing measures are in effect, typically concurrent with a high number of infections. They reduce protective behaviour when vaccination coverage is high or when mandated contact reduction measures are relaxed, typically concurrent with a reduction of infections. We demonstrate that waning of infection and vaccine-induced immunity are important for reproducing disease transmission in autumn 2021.

## 1. Introduction

Coronavirus disease 2019 (COVID-19) has been a global challenge leading to millions of infections and thousands of deaths globally. Before the availability of vaccines, most countries relied solely on the implementation of a range of non-pharmaceutical interventions (NPIs) such as partial closings of businesses, lockdowns and mask-wearing to curb the spread of SARS-CoV-2 and avoid overburdening healthcare systems [1–3]. With the development of COVID-19 vaccines, policy makers started vaccination campaigns with the aim to protect individuals and relax NPIs. Vaccines became the most important intervention for mitigating disease severity and spread, allowing the return of social and economic activities [4–8].

Human behaviour plays an important role on the efforts to control the transmission of COVID-19, since the effectiveness of mitigation measures depends on NPI compliance and vaccine acceptance. People are most likely to adopt protective behaviour when mortality or the perception of risk is high, and resume normal life as the perceived risk declines [9–11]. Hence, it is crucial to consider the effects of behaviour change over time so that the design of effective infection mitigation policies can be achieved.

Since the onset of the COVID-19 pandemic, many studies have developed mathematical models to describe the dynamics of transmission of the disease [12–14]. Many of the proposed models are extensions of the classical Kermack–McKendrick susceptible–infectious–recovered (SIR) epidemic model [15], which predicts the number of individuals who are susceptible to infection, actively infected, or have recovered from infections at any given time [16]. Several studies have extended the SIR model by considering additional compartments to account for asymptomatic cases, hospitalizations, quarantine, vaccination, disease-induced death and/or heterogeneity of the population [17]. These epidemic models can also be coupled with models describing behaviours that are affected by and affect the disease transmission dynamics [18,19]. Some of the proposed COVID-19 compartmental models have considered how individuals respond to the disease dynamics and how the disease dynamics are affected by these behavioural responses [20–30].

In this study, we extend a compartmental SEPIR model first published by Moyles *et al.* [20]. The model divides the population into five possible disease states: susceptible (*S*), exposed (*E*), pre-symptomatic (*P*), infected (*I*) (both symptomatic *I*_*S*_ and asymptomatic *I*_*A*_) and recovered (*R*). It also includes three classes of social distancing over each disease state. Additionally, infections are delineated into those that are known and unknown. The model was used to study the first several months of the COVID-19 pandemic and NPI compliance in Ontario, Canada. However, Moyles *et al.* [20] did not include vaccination, waning immunity or viral variants, as these were concerns after publication of their work. The main purpose of our study is to adapt their model to include vaccination, which confers some immunity to the disease, and waning from all sources of immunity. The effects of waning immunity have been incorporated in some epidemiological models of the COVID-19 pandemic [8,31–35]. Furthermore, we extend the model to include variants of concern by allowing modification of the transmissibility of the disease over time. We do not include the Omicron variant in our study since data acquisition became more difficult as governments reduced testing and started lifting NPIs.

To the best of our knowledge, no previous studies have examined the coupled effects of dynamic social distancing and cost-based relaxation, waning immunity, vaccination and new variants of concern on the progression of the pandemic. Our study is organized as follows. In §2.1, we introduce the extended SEPIR model including new parameters, values for which are derived from existing literature or fit to data from Public Health Ontario (PHO) [36,37]. We then present the estimated parameters using time horizons of public policy implementations in Ontario developed by Dick *et al.* [38]. Additionally, we investigate the effect of waning immunity. We compare our results with publicly accessible data on positivity rate, daily incidence and seroprevalence [36,39]. Following Moyles *et al.* [20], we focus our work on the Canadian province of Ontario. We discuss the conclusions of our work in §4.

## 2. Methods

### 2.1. Susceptible–exposed–pre-symptomatic–infected–recovered model

**Figure 1.**
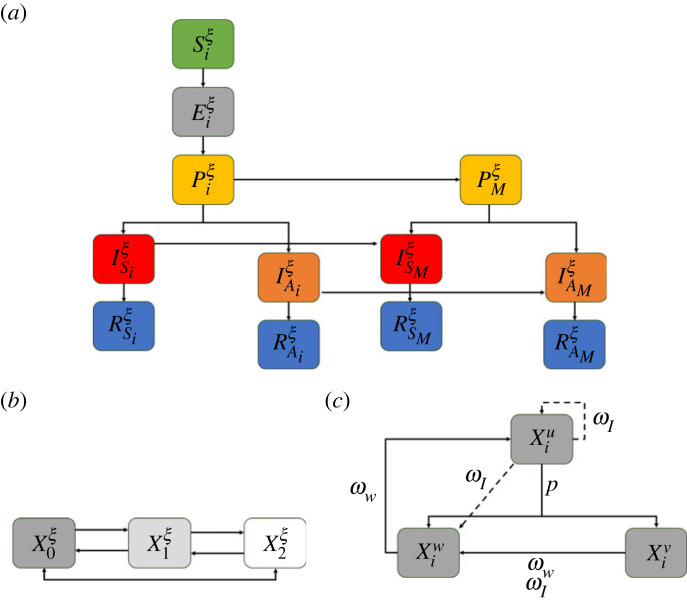
Schematic of the disease progression through susceptible–exposed–pre-symptomatic infectious–infectious asymptomatic–infectious symptomatic–recovered (SEPIR) (*a*). Each compartment, *X*, can have one of three levels of social distancing, from no social distancing (subscript 0) to full isolation (subscript 2) (*b*). Individuals in each compartment begin in an unvaccinated class (superscript *u*) and can receive a vaccination at rate *p* (except those in *I*_*S*_). This vaccination either confers immunity (superscript *v*) or it does not (superscript *w*) (*c*). Protection from the vaccination or infection can wane with rates *ω*_*V*_ and *ω*_*I*_, respectively, while perception of immunity can wane at rate *ω*_*W*_. Known infections (via testing) are shown with subscript *M* and are isolated from the population until they have lost protection. (*a*) Disease progression, (*b*) contact reduction and (*c*) waning protection.

We developed a compartmental model based on one proposed by Moyles *et al.* [20] depicted in figure 1*a*,*b*. Briefly, panel (*a*) shows disease progression from susceptible (*S*) to recovered (*R*) through the different infection stages: non-infectious (*E*), pre-symptomatic infectious (*P*), asymptomatic infectious (*I*_*A*_), and symptomatic infectious (*I*_*S*_). Panel (*b*) illustrates movement between three social distancing classes, with subscripts 0, 1 and 2 denoting no social distancing, some social distancing and complete isolation, respectively. Individuals can move up and down the social distancing ladder; however, the disease progression pathway shown in (*a*) is the same for all individuals in different social distancing states. Movement between social distancing classes is allowed unless infection status is known, at which point the individual is removed and quarantined.

We extend the model of Moyles *et al.* to include vaccine-induced immunity and perceived immunity (each with waning), as shown in (*c*). Superscripts in (*c*) illustrate the immunity class of individuals between unvaccinated (*u*), vaccinated (*v*) or with perceived immunity (*w*). Individuals who are not symptomatic or who have not tested positive vaccinate at a rate *p* transitioning them from the *u* state to either *v* or *w* depending on the success of the vaccine denoted *q*^*v*^. The model does not explicitly consider modelling the immune system and as such we do not quantify levels of immunity through multiple doses; individuals are either vaccinated or are not. An individual recruited into the *v* state via successful vaccination loses that protection at a wanting rate *ω*_*V*_. Those in the perceived immunity class, *w*, transition to *u* at a rate *ω*_*W*_. Therefore, within the context of this model, when someone wanes their perceived immunity they are once again classified as unvaccinated. When an individual becomes infected, they eventually transition to a recovery class which provides natural immunity which we assume wanes at a rate immunity rate *ω*_*I*_.

All infections transition from a susceptible state through to recovery but with rates and probabilities dependent on the immunity and social distancing status. As such, we denote our variables $X_{i}^{\xi }$ where *X* ∈ {*S*, *E*, *P*, *I*_*S*_, *I*_*A*_, *R*_*S*_, *R*_*A*_} is the disease state, the subscript *i* ∈ {0, 1, 2} is the physical distancing level, and the superscript *ξ* ∈ {*u*, *v*, *w*} indicates immunity status. The *M* subscript in figure 1*a* indicates those who have tested positive for the virus and are isolated from the population until recovery. We summarize each of the model disease classes as follows:

— Susceptible individuals denoted by $\left(S{\left(t\right)}_{i}^{\xi }\right)$, who are eligible to be infected by the pathogen;
— exposed individuals denoted by $\left(E{\left(t\right)}_{i}^{\xi }\right)$, who have been infected but are incubating the virus; they are not transmissible and have a low enough viral load that they would not test positive for COVID-19;
— pre-symptomatic individuals denoted by $\left(P{\left(t\right)}_{i}^{\xi }\right)$, who are infectious but have not had the disease long enough to show symptoms;
— infected-symptomatic individuals denoted by $\left(I_{S}{\left(t\right)}_{i}^{\xi }\right)$ who are infectious and have started showing symptoms;
— infected-asymptomatic individuals denoted by $\left(I_{A}{\left(t\right)}_{i}^{\xi }\right)$, who are infectious and never show symptoms;
— removed-symptomatic individuals denoted by $\left(R_{S}{\left(t\right)}_{i}^{\xi }\right)$, who were symptomatic, but are no longer infectious;
— removed-symptomatic individuals denoted by $\left(R_{A}{\left(t\right)}_{i}^{\xi }\right)$, who were asymptomatic, but are no longer infectious;

with *t* as time in days since the onset of the pandemic, taken here to be 10 March 2020. A summary of the immune status of each compartment is in table 1. All compartments sum to the total population of Ontario, *N* = 13 448 494, which is constant in time as we do not consider recruitment from birth or death. The governing differential equations for the full model depicted in figure 1 are detailed in appendix A. Table 2 defines the parameters used throughout this study in simulating the model equations in appendix A. Parameters listed as *fit* are estimated following §2.4. It is possible, in general, to become infected after vaccination and ultimately recover (e.g. $R_{S_{i}}^{v}$ and $R_{A_{i}}^{v}$). We do not consider any form of additional immunity conferred having both vaccination and infection. Furthermore, when immunity wanes, we assume it is at the natural immunity waning rate *ω*_*I*_ and that both natural and vaccinated immunity is lost concurrently so that the individual is fully susceptible again. All individuals in the $X_{i}^{u}$ class are naive to their infection status (except those who have tested positive) and therefore when they become infected and wane their immunity, it is assumed that they transition to $S_{i}^{u}$. Those who test positive or those who receive a vaccination have some awareness of their immunity or perceived immunity. Therefore, when their immunity wanes, we assume they transition to $S_{i}^{w}$. When someone with a vaccination becomes infected, we assume that the infection will progress to completion, i.e. there will be no waning of vaccine protection or otherwise until recovery.

**Table 1.** The table shows what type of immunity each compartment has with $N^{\xi }=[S^{\xi },E^{\xi },P^{\xi },P_{M}^{\xi },I_{S}^{\xi },I_{S_{M}}^{\xi },I_{A}^{\xi },I_{A_{M}}^{\xi }]$ and $R^{\xi }=[R_{S}^{\xi },R_{A}^{\xi },R_{S_{M}}^{\xi },R_{A_{M}}^{\xi }]$, where *ξ* ∈ {*u*, *v*, *w*}.

classes	no immunity	infection-induced immunity	vaccine-induced immunity	perceived-induced immunity
$N^{u}$	✓			
$R^{u}$		✓		
$N^{v}$			✓	✓
$R^{v}$		✓	✓	✓
$N^{w}$	✓			✓
$R^{w}$		✓		✓

**Table 2.** Parameter definitions and values for model in figure 1 and appendix A.

	definition	value	comment
*N*	population of Ontario	13 448 494	2016 census
*N*_crit_	critical population at which healthcare resources are overwhelmed	81 301	[20]
*R*_0_	basic reproduction number	2.4	[20]
*β*	transmission rate of wild-type strain	0.223 d^−1^	[20]
*δ*	reduction in transmission due to social distancing in class 1	0.25	[20]
*α*	reduction in transmission due to being asymptomatic	0.5	[20]
*σ*	rate at which exposed class enter pre-symptomatic class	2 d^−1^	[20]
*ϕ*	rate at which pre-symptomatic class can being showing symptoms	0.2174 d^−1^	[20]
*Q*	proportion of infected individuals who show symptoms	0.69	[20]
*γ*	rate at which an infected person is no longer infectious	0.1 d^−1^	[20]
*μ*_max_	maximal rate at which someone in the *u* immunity class moves from a less socially distant class to a more socially distant class	1 d^−1^	[20]
*ν*_max_	maximal rate at which someone in the *u* immunity class moves from a more socially distant class to a less socially distant class	1 d^−1^	[20]
*μ*_*I*_	rate at which people showing symptoms choose to isolate	0.01 d^−1^	[20]
*q*_0_	proportion of *S*_0_ socially distancing into *S*_1_	0.9	[20]
*q*_2_	proportion of *S*_2_ relaxing social distancing into *S*_1_	0.6	[20]
*q*_*I*_	proportion of symptomatic individuals $I_{S_{0}}$ who isolate into $I_{S_{1}}$	0.6	[20]
$\rho_{A}^{u}$	testing rate for someone in the *u* immunity class not showing symptoms to test positive		fit
$\rho_{S}^{u}$	testing rate for someone in the *u* immunity class showing symptoms to test positive	$4\rho_{A}^{u}$	[20]
*M*_*c*_	critical active cases to induce social distancing		fit
*M*_0_	active cases that lead to half the maximal rate of social distancing	2*M*_*c*_	[20]
*K*_*c*_	critical approximate disease doubling rate to induce social distancing		fit
*K*_0_	approximate disease doubling rate that leads to half the maximal rate of social distancing	4*K*_*c*_	[20]
*C*_*c*_	critical cost to induce social relaxation	50	[20]
*C*_0_	cost that leads to half the maximal rate of social relaxation	100	[20]
*p*	vaccination rate for someone in immune status *u* to go to *v* or *w*		fit
*q*^*v*^	proportion of vaccinated individuals obtaining vaccine-induced immunity	1	chosen
$\mu_{\max}^{v}$	maximal rate at which someone in the *v* or *w* immunity class moves from a less socially distant class to a more socially distant class	0.5*μ*_max_	chosen
$\nu_{\max}^{v}$	maximal rate at which someone in the *v* or *w* immunity class moves from a more socially distant class to a less socially distant class	2*ν*_max_	chosen
$\rho_{A}^{v}$	testing rate for someone in the *v* or *w* immunity class not showing symptoms to test positive	0.5*ρ*_*A*_	chosen
$\rho_{S}^{v}$	testing rate for someone in the *v* or *w* immunity class showing symptoms to test positive	$4\rho_{A}^{v}$	chosen
ϵ	modified transmission rate from successful vaccination	0	chosen
*ω*_*I*_	waning rate of induced immunity from infection	0.005 d^−1^	chosen
*ω*_*V*_	waning rate of induced immunity from vaccination	0.005 d^−1^	chosen
*ω*_*W*_	waning rate of perceived immunity	0.07 d^−1^	chosen

### 2.2. COVID-19 testing

In this study, we compare the number of cumulative and active reported infections, seroprevalence, daily incidence and positivity rate calculated by our model with the data provided by Public Health Ontario.

#### 2.2.1. Active reported infections

Active reported infections, *M*_*A*_ are defined by the sum of reported pre-symptomatic, asymptomatic and symptomatic cases with different immunity levels who have not yet recovered, i.e. would not yield a negative test result. We define them as

2.1 $$M_{A}=\sum_{\begin{array}{c} \xi \in \{u,v,w\}\\ i\in \{0,1,2\} \end{array}}(P_{M_{i}}^{\xi }+I_{S_{M_{i}}}^{\xi }+I_{A_{M_{i}}}^{\xi }).$$

Note that we assume that all reported infections will fully isolate.

#### 2.2.2. Cumulative reported infections

We define *M* to be the cumulative newly reported cases. We define the rate of change of cumulative reported incidence as a sum of pre-symptomatic, asymptomatic and symptomatic infections who have tested positive at time *t* as follows:

2.2 $$\dot{M}=\sum_{\begin{array}{c} \xi \in \{u,v,w\}\\ i\in \{0,1,2\} \end{array}}(\rho_{s}^{\xi }I_{S_{i}}^{\xi }+\rho_{a}^{\xi }(P_{i}^{\xi }+I_{A_{i}}^{\xi })),$$

where we define the testing rate of symptomatic infections to be $\rho_{s}^{\xi }$ and for asymptomatic infections to be $\rho_{a}^{\xi }$, *ξ* ∈ {*u*, *v*, *w*}. From table 2, we note that $\rho_{i}^{w}=\rho_{i}^{v}$ since those in the *w* class perceive to have immunity and therefore are assumed to behave as if immune.

#### 2.2.3. Total vaccination administered

Cumulative vaccination, *V*_*A*_, is the total vaccines administered. The rate of change here is defined by the sum of all eligible vaccine recipients who vaccinate at time *t* with rate *p* and is defined as

2.3 $${\dot{V}}_{A}=\sum_{i=0}^{2}p(S_{i}^{u}+E_{i}^{u}+P_{i}^{u}+I_{A_{i}}^{u}+R_{A_{i}}^{u}).$$

Importantly, we assume that those who have symptomatic infection, have recovered from symptomatic infection, or have tested positive for having an infection are ineligible to receive a vaccine.

#### 2.2.4. Seroprevalence

Serology testing, which tests someone’s blood to see if they have antibodies for COVID-19, is used as a measure of population-level infection and immunity. Seroprevalence, *S*_*R*_, is estimated by the number of people who test positive for COVID-19 antibodies based on serology data. Herein, we assume that people with COVID-19 antibodies will belong to the recovered class and thus

2.4 $$S_{R}=\sum_{\begin{array}{c} \xi \in \{u,v,w\}\\ i\in \{0,1,2\} \end{array}}(R_{S_{i}}^{\xi }+R_{A_{i}}^{\xi }+R_{S_{M_{i}}}^{\xi }+R_{S_{A_{i}}}^{\xi }).$$

We note that individuals that wane out of the recovered classes will not have positive serology tests in our model.

#### 2.2.5. Daily reported infection incidence

*D*_*I*_ refers to the number of newly diagnosed COVID-19 cases per day, and is defined as

2.5 $$D_{I}=M(t)-M(t-1).$$

#### 2.2.6. Positivity rate

Since we assume that all individuals are eligible for testing, then we can define the test positivity rate as the number of positive tests (daily incidence) divided by total tests administered across the entire population. Since asymptomatic individuals are unaware they are infectious prior to a positive test result, we assume that the testing rate for the general population is the same as that for asymptomatic individuals, $\rho_{a}^{\xi }$, *ξ* ∈ {*u*, *v*, *w*}. Thus, we define the total tests *T*_*T*_

$$T_{T}=\sum_{\begin{array}{c} \xi \in \{u,v,w\}\\ i\in \{0,1,2\} \end{array}}[\rho_{a}^{\xi }(S_{i}^{\xi }(t)+E_{i}^{\xi }(t)+P_{i}^{\xi }(t)+I_{A_{i}}^{\xi }(t)+R_{S_{i}}^{\xi }(t)+R_{A_{i}}^{\xi }(t))+\rho_{s}^{\xi }I_{S_{i}}^{\xi }],$$

and the test positivity rate

$$\rho^{+}:=\frac{D_{I}}{T_{T}}.$$

### 2.3. Physical distancing functions

We model the transition between the different social distancing classes as in [20], by assuming that individuals who are not vaccinated move from social distancing class 0 to class 1 with rate *μ*^*i*^ given by

2.6 $$\mu^{i}=\mu_{\max}^{i}\left(\frac{{\left[K_{M}-K_{c}\right]}_{+}}{{\left[K_{M}-K_{c}\right]}_{+}+K_{0}-K_{c}}\right)\left(\frac{{\left[M_{A}-M_{c}\right]}_{+}}{{\left[M_{A}-M_{c}\right]}_{+}+M_{0}-M_{c}}\right),$$

where $\mu_{\max}^{i}$ is the maximal rate of social distancing, ${\left[\cdot \right]}_{+}=\max(\cdot ,0)$, *M*_*A*_ are the active cases given by (2.1), and *K*_*M*_ is the doubling rate given by

$$K_{M}=\frac{dM/dt}{M\ln(2)}.$$

The parameters *M*_*C*_ and *K*_*C*_ represent critical values for *M*_*A*_ and *K*_*M*_ respectively to activate social distancing. If *M*_*A*_ < *M*_*C*_ or *K*_*M*_ < *K*_*C*_ then *μ*^*i*^ ≡ 0. The parameters *M*_0_ and *K*_0_ represent values of *M*_*A*_ and *K*_*M*_, respectively, where the social distancing function *μ*^*i*^ transitions from linear growth to a saturated level with rate $\mu_{\max}^{i}$. We note that the superscript *i* ∈ {*u*, *v*, *w*} in *μ*^*i*^ indicates that behaviour, specifically the maximum rate, depends on vaccine status. We assume that individuals who are vaccinated are slower in transitioning from social distancing class 0 to 1 by setting *μ*^*v*^ = *μ*^*w*^ = *μ*^*u*^/2. We assume that individuals transition from social distancing class 1 to class 2 with rate *μ*^*i*^/2 to take into account that people who have already reduced their contacts will be slower in fully isolating.

Individuals decrease social distancing based on some cost, *C*, with rate *ν* defined as in [20],

2.7 $$\nu^{i}=\nu_{\max}^{i}\left(\frac{{\left[C-C_{c}\right]}_{+}}{{\left[C-C_{c}\right]}_{+}+C_{0}-C_{c}}\right),$$

where $\nu_{\max}^{i}$ is the maximal rate at which physical distancing can be relaxed. Similarly to *μ*^*i*^, the superscript indicates vaccine status and we assume that vaccinated individuals relax faster than unvaccinated so that *ν*^*v*^ = *ν*^*w*^ = 2*ν*^*u*^. *C*_*c*_ is the cost that individuals are willing to bear before relaxing their behaviour (if *C* < *C*_*C*_ then *ν*_*i*_ ≡ 0), and *C*_0_ is the transition between linear growth of *ν*^*i*^ and a saturated relax state at $\nu^{i}=\nu_{\max}^{i}$.

For the cost of social distancing, *C*, those in all immunity groups who are susceptible or exposed (i.e. would not test positive for the virus) and fully isolating (social distance level 2) endure the maximal cost, as they are unnecessarily reducing their contacts. This maximal cost has many components including direct financial cost from missing work as well as psychological cost from isolating, and therefore, it is difficult to assign a direct dollar amount. Following Moyles *et al.* in [20], we scale *C* to be in days, where 1 day represents the equivalent of the entire population being in the fully isolated state. Thus, at any point in time, *t*, necessarily *C* ≤ *t*. Individuals who are susceptible or exposed in social distancing class 1 reduce their contacts by *δ* and thus we assume they have a reduced cost compared with the full isolating group of (1 − *δ*) (as group 1 reduces contacts more, i.e. *δ* approaches 0, their cost approaches that of the fully isolating group). Overall, following Moyles *et al*. in [20], we define the following for cost,

2.8 $$\dot{C}=\frac{N_{\mathrm{Crit}}}{N}\sum_{\xi \in \{u,v,w\}}((S_{2}^{\xi }+E_{2}^{\xi })+(1-\delta )(S_{1}^{\xi }+E_{1}^{\xi })),$$

with the summation accounting for the different immunity status, something that was absent from the original model. The parameter *N*_crit_ is the population above which hospital resources are strained.

### 2.4. Parameter values and estimation

In this study, we estimate parameters (i) *K*_*c*_: critical approximate disease doubling rate to induce social distancing, (ii) *M*_*c*_: critical active cases to induce social distancing, and (iii) *ρ*_*a*_: testing rate for asymptomatic person to test positive as these parameters are assumed to vary within different public health mitigation periods. Additionally, we estimate *p*, the rate of vaccination. For parameter fitting, we use data from Public Health Ontario [36,37] on cumulative and active reported cases, and total vaccines administered from 10 March 2020 to 30 November 2021. Since we allow immunity to wane, then an individual can return to an ‘unvaccinated’ status again. Therefore, we should separate individuals with active vaccine protection from those who have been administered a vaccine (*V*_*A*_ from (2.3)) as it is only the latter group for which there is data. However, our model structure theoretically allows people to re-vaccinate once they are in the *u* state again, something that was generally not permissible in Ontario after receiving the primary series. As such, we use everyone in immunity state *v* and *w* as a measure of vaccine count as opposed to vaccines administered to deal with this double-counting effect.

We estimate the parameters in different time windows defined by the time period over which certain policies were in effect to investigate how their values change based on NPIs and pharmaceutical interventions. We choose the date and the category of the implemented NPI as developed by Dick *et al.* [38] where the authors used government resources and creditable news agencies to provide the timeline of categorized public health interventions from 12 March 2020 to 5 January 2022. In table 3, we provide the dates of each time window and the corresponding policy.

**Table 3.** Estimated values of *K*_*c*_, *M*_*c*_, *ρ* and *p* for each time window. The row colour indicates the dominant variant assumed to be circulating at that time with no colour being wild-type (transmissibility *β*), yellow being the Alpha variant B.1.1.7 (transmissibility 1.5*β*) and green being the Delta variant B.1.617.2 (transmissibility 2*β*).

time window	important dates		rationale	*K*_*c*_	*M*_*c*_	*ρ*_*a*_	*p*
1	10 Mar 2020	7 Jun 2020	lockdown and gradual reopening	0.0635	0.0239	0.0094	—
2	7 June 2020	20 Aug 2020	stage 2 and 3 mosaic	0.0635	0.0239	0.0061	—
3	20 Aug 2020	25 Dec 2020	tightening Measures, second wave	0.0160	0.0000	0.0021	—
4	25 Dec 2020	19 Jan 2021	tightening Measures, second wave	0.0000	0.2885	0.0024	—
5	19 Jan 2021	28 Jan 2021	stay at home	0.0000	0.0000	0.0024	—
6	28 Jan 2021	15 Feb 2021	stay at home	0.0031	0.0000	0.0038	—
7	15 Feb 2021	12 Mar 2021	reopening scenarios	0.0048	0.0000	0.0040	0.0011
8	12 Mar 2021	4 Apr 2021	reopening scenarios	0.0070	0.0000	0.0020	0.0000
9	4 Apr 2021	9 May 2021	emergency stay at home	0.0000	0.3312	0.0024	0.0002
10	9 May 2021	10 June 2021	emergency stay at home	0.0000	0.0000	0.0029	0.0016
11	10 June 2021	15 June 2021	S1	0.0096	0.0097	0.0040	0.0187
12	15 June 2021	29 June 2021	S1	0.0096	0.0097	0.0043	0.0153
13	29 June 2021	15 July 2021	S2	0.0096	0.0097	0.0010	0.0288
14	15 July 2021	1 Sep 2021	S3	0.0096	0.0097	0.0003	0.0221
15	1 Sep 2021	30 Nov 2021	S3	0.0096	0.0097	0.0020	0.0230

To start the parameter fitting, we initialize the parameters *K*_*c*_, *M*_*c*_, *ρ*_*a*_ and *p* with random values in the first time window and then employ a nonlinear least-squares method to find the values of the parameters so that the simulated cumulative and active reported cases, and total number of vaccinations, best fit the data. For subsequent windows, we initialize parameters with the value obtained in the previous time window before once again proceeding with the least-squares algorithm. We take into account that the emergence of SARS-CoV-2 variants can affect the transmission rate of the disease. We define *β* as the transmission coefficient for the wild-type strain which was dominant from the start of COVID-19 until the middle of February 2021. Between 15 February and 29 June 2021, the Alpha variant (B.1.1.17) was dominant. For fitting parameters in this window, we modify the transmission coefficient to 1.5*β* accounting for the higher reproduction number of this variant [40]. Similarly, from 29 June 2021 to 31 December 2021 the Delta variant (B.1.617.2) was dominant and we modify the transmission to 2*β* [40].

### 2.5. Initial conditions

We initialize all compartments to be zero except for the symptomatic infectious $I_{S}^{0}$ and the susceptible *S*^0^, assuming that $I_{S}^{0}(t=0)=0.0002N$ and *S*^0^(*t* = 0) = 0.9998*N*, where *t* = 0 is the initial time, 10 March 2020.

### 2.6. Sensitivity analysis

We perform a sensitivity analysis on the waning parameters *ω*_*I*_, *ω*_*V*_ and *ω*_*W*_. To do so we generate 1000 samples of the parameters *ω*_*I*_, *ω*_*V*_ and *ω*_*W*_ using the Latin hypercube method [41]. We assume that the quickest vaccine-induced immunity, *ω*_*V*_, or infection-induced immunity, *ω*_*I*_, can wane is four months and the slowest is 2 years [35]. The quickest the perceived induced immunity, *ω*_*W*_ , can wane is four months, and the slowest is 1 year [42]. We did not test the sensitivity of our model to disease-related parameters since our model is an extension of the model presented in [20] and the authors carried out sensitivity analysis on disease parameters there.

## 3. Results

### 3.1. Time windows

We provide the fitting results for each time window in table 3 where rows are colour-coded according to the dominant variant and hence which transmission coefficient is used. Parameters that are not fit are held constant in each window and provided in table 2.

From the start of the pandemic until 20 August 2020 (time windows 1 and 2), the values of the parameters *K*_*c*_ and *M*_*c*_ remain the same, indicating that individuals had the same level of vigilance during that time period, while the testing rate *ρ*_*a*_ is high during the first time window, but it decreases during the second time window. The decrease in the values of *K*_*c*_ and *M*_*c*_ between 20 August and 25 December 2020 shows that individuals became more cautious, while testing decreases further compared with the previous time period. During that time window, more strict measures were implemented in Ontario, explaining the increased vigilance. During time window 4, we observe that the value of *K*_*c*_ drops, but the values of *M*_*c*_ and *ρ*_*a*_ increase. Increases in the value of *M*_*c*_ indicate that more cases are needed to induce social distancing, but the critical doubling rate is zero, meaning that any increase in the doubling rate leads to more vigilance. Increases in the value of *M*_*c*_ and reduction in the value of *K*_*c*_ might occur during the exponential phase of spread of the disease when the number of cases might not be high, but the doubling rate is high and individuals are more cautious knowing that the number of cases is exponentially growing. From 19 January to 15 February 2021 (time windows 5 and 6), a stay-at-home order was in effect in Ontario, and this resulted in people increasing social distancing as the value of *M*_*c*_ remains zero, implying that any number of cases triggers social distancing. From 15 February to 4 April 2021 (time windows 7 and 8), although the government was considering relaxation of the mitigation measures, the values of *M*_*c*_ remain zero showing that individuals were still vigilant and continue to social distance if the number of cases is non-zero. During time windows 9 and 10, the stay-at-home order was again in effect. Although, the value of *M*_*c*_ increased during time window 9, the value of *K*_*c*_ remains zero for both time windows, indicating that people are reducing their contacts if the doubling rate is greater than zero. Finally, from 10 June to 30 November 2021 (time windows 11 to 15), the values of *K*_*c*_ and *M*_*c*_ remain constant and increase compared with the time period between 9 May and 15 June. It is possible that the increase in vaccine coverage resulted in people being more relaxed about social distancing, and would reduce their social activities only when the doubling rate or number of cases would surpass the value of *K*_*c*_ and *M*_*c*_, respectively.

### 3.2. Waning immunity

**Figure 2.**
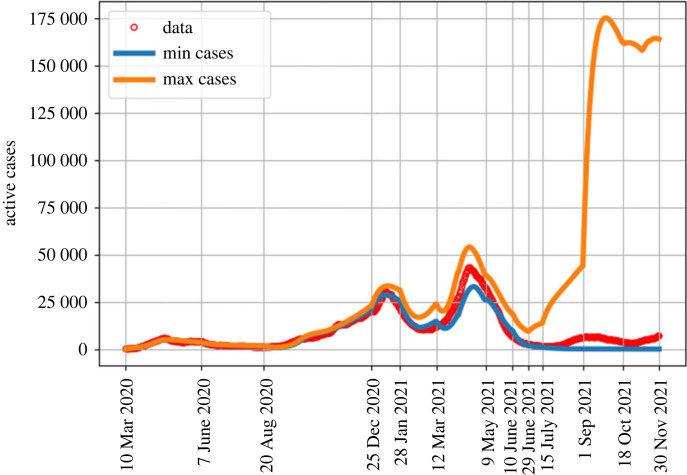
The minimum and maximum number of active cases per day, obtained by numerically solving the model equations, for different values of ϵ, *ω*_*I*_, *ω*_*V*_ and *ω*_*W*_.

In figure 2, we present the results from our sensitivity analysis based on the 1000 samples of the parameters *ω*_*I*_, *ω*_*V*_ and *ω*_*W*_ and the estimated values of *K*_*c*_, *M*_*c*_, *ρ* and *p*. We plot the trajectory that corresponds to the minimum number of active cases at each time (blue curve in figure 2) as well as the trajectory that leads to the maximum number of active cases at each time (orange curve in figure 2). All other trajectories in the 1000 samples were contained within the envelope between these two curves. The minimum trajectory is generated from slow waning times that are near 2 years while the maximum trajectory is generated from fast waning times that are near four months. We observe that the minimum and maximum number of daily active cases are similar in magnitude up to 25 December 2020, which implies that model predictions are not affected by the waning immunity parameters up to this date. Following the minimum trajectory after 25 December 2020, the model predicts no active infections after 29 July 2021 meaning that without sufficient waning, the model does not capture the fourth wave. Conversely, following the maximum trajectory, we see an overestimation of cases, particularly after 29 July 2021. This indicates that a moderate waning rate such as that selected in table 2 is appropriate for active case levels recorded in autumn 2021.

### 3.3. Model prediction versus observed data

**Figure 3.**
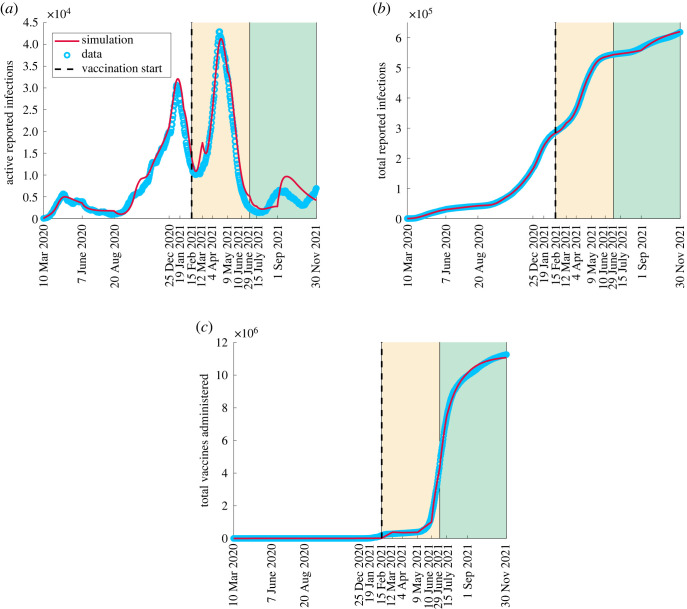
Comparison between model simulations and data from Public Health Ontario between 10 March 2020 and 30 November 2021. Model simulations are fit to data from [36] in (*a*) and (*b*) for active and total reported cases, respectively. Simulations are fit to data from [37] in panel (*c*) for vaccines administered. The white patch indicates the time over which the wild-type strain was dominant (transmission coefficient *β*), the yellow patch indicates the time over which the Alpha B.1.1.7 variant was dominant (transmission coefficient 1.5*β*), while the green patch indicates the time over which the Delta B.1.617.2 variant was dominant (transmission coefficient 2*β*). The vertical dashed line indicates the vaccination starting date. (*a*) Active reported cases, (*b*) total reported cases, (*c*) total vaccines administered.

The results from simulating the model following the fitting of *K*_*c*_, *M*_*c*_, *ρ*_*a*_ and *p* are illustrated in figure 3. Along with simulations, we plot data on active reported infections (figure 3*a*), cumulative reported infections (figure 3*b*), and the total vaccines administered (figure 3*c*) [36,37]. We observe a satisfactory model prediction of observed data for cumulative incidence and total vaccination administered criteria between 10 March 2020 and 30 November 2021. For active reported infections, the fit is satisfactory until approximately September 2021 after which there is an overshoot compared with the data.

**Figure 4.**
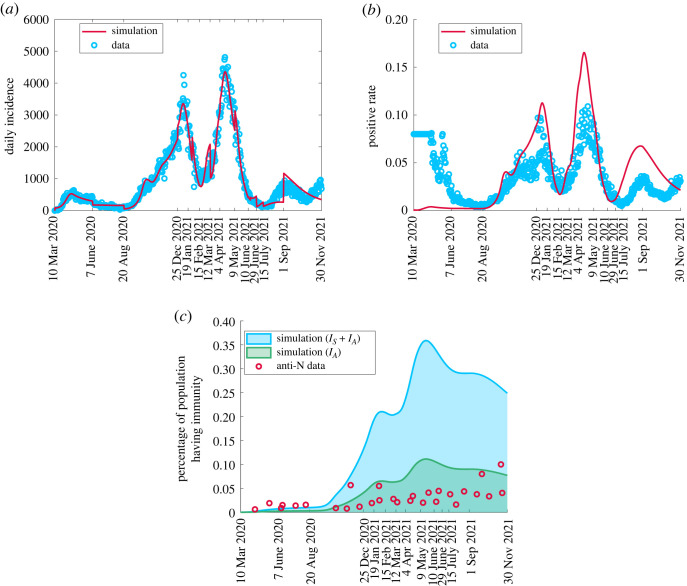
The simulation results for the daily incidence, positivity rate, seroprevalence have been projected with health data from 10 March 2020 to 30 November 2021. Daily incidence and test positivity data are provided from Public Health Ontario [36,37]. Seroprevalence data are from acquired infection through a serology test on the anti-nucleocapsid (anti-N) [39]. (*a*) Daily incidence, (*b*) test positivity rate, (*c*) seroprevalence.

To assess the robustness of our estimates, we compare the simulated model results with data which was not used to fit the model, particularly daily incidence (figure 4*a*), test positivity rate (figure 4*b*) and seroprevalence (figure 4*c*). We observe a relatively good agreement between data and simulation for daily incidence and positivity rate, with the largest discrepancy in testing rate concurrent with the deviation in matching simulated active cases to data.

The estimated seroprevalence is higher than the data suggests, although a good agreement is observed if only asymptomatic cases are considered (green shaded region figure 4*c*). The data on seroprevalence in figure 4*c* are based on studies from blood donors aged 16+ from Canadian Blood Services (CBS) [39,43] acquiring infection via the presence of anti-nucleocapsid (anti-N) in a serology test. We note here that our model does not distinguish between serology and T-cell-mediated immunity. It is possible for individuals to have T-cell immunity even when antibody levels have waned. A recent study reported that high T-cell memory levels can protect against COVID-19 infection [44]. An additional reason for discrepancy could be that our model presents immunity from the entire population irrespective of age whereas the CBS serological testing is conducted in ages 16+ only. The inclusion of age structure may reduce our seroprevalence estimates as the disease has been shown to present differently in children and severity of disease can affect the profile of antibody response [45,46]. However, even with age-structuring Dick *et al.* estimated seroprevalence higher than suggested by the data, although less of a difference than we see here [35].

**Figure 5.**
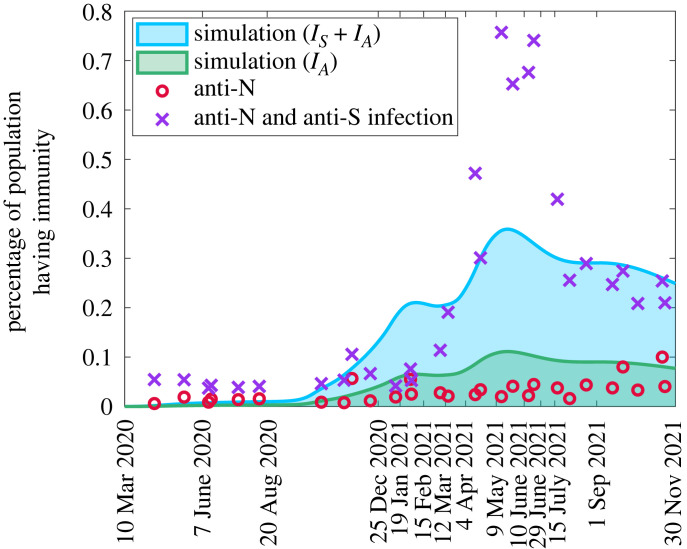
Comparison between simulated seroprevalence and serology data from Canadian Blood Services [39] for both anti-N as well as anti-N plus infection-induced anti-S.

We can improve agreement with seroprevalence by reconsidering the data comparison. Serology tests from CBS also identify anti-spike (anti-S) which can be acquired through infection or induced through vaccination. In figure 5, we plot the simulated data and anti-N seroprevalence similar to figure 4*c* but add the anti-S data solely from infection. We estimate this by comparing the time series of anti-S with the administered vaccines in figure 3*c*. Since our model confers immunity directly when vaccination happens, then the administered vaccines should provide a proxy for induced anti-S from vaccination. We assume that the difference between the administered vaccines and anti-S serology data represents the amount of anti-S attributed to infection. Including this additional serological information, we see a much stronger agreement between seroprevalence data and our simulations. Using this new data, our simulations provide an underestimation of seroprevalence at the peak, but importantly we see the correct qualitative trends with agreement at the peak time and the onset of waning.

## 4. Discussion

In this work, we proposed a compartmental model coupling the effects of dynamic social distancing, cost-based relaxation, different immunity levels, vaccination and new variants of concern to study and recreate the history of the COVID-19 pandemic up to December 2021 in Ontario. The model can fairly accurately predict different quantities of interest including active cases, vaccination, daily incidence and positivity rate. However, when comparing our model with anti-N seroprevalence data, we saw a large overestimation. This was significantly improved when we also included infection-acquired anti-S measurements. Our model assumptions limit the accuracy we may get in seroprevalence estimates. As mentioned, we neglect age structure which can play an important role in the disease profile. We also do not distinguish people who will test sero-negative for antibodies but are immune to infection. This may be of particular importance in mild infections where the presence of antibodies may exhibit a shorter duration [46]. We do not include death in our model, which means that those individuals who die from severe disease are still qualified as recovered in our model and therefore would contribute to a positive seroprevalence. However, death only accounts for 1% of total cases and may not be a significant impact [47].

We concluded that if we assume that it takes 2 years for disease- or vaccine-induced immunity to wane, our model does not capture the fourth wave in Ontario. Our sensitivity analysis showed that waning immunity would not change anything in the model predictions on active cases until 25 December 2020. However, the model is more sensitive after 25 December 2020, and the values of the waning parameters affect the fitting results. Our simulations and sensitivity analysis showed that waning immunity is crucial to capture more accurately the disease dynamics and predict multiple waves over a long time period. In future work, we want to extend the time period to include the Omicron variant and study the effect of evading immunity on disease dynamics.

We estimated key parameters affecting the vigilance of individuals at different time windows and found that NPIs influence how they increase or decrease their contacts. For example, individuals are more cautious when stricter measures are introduced such as stay-at-home orders or lockdowns. Our results also indicate that people started being more relaxed about social distancing after May 2021, which is approximately when vaccine coverage increased in Canada. This shows the importance of having a model that incorporates dynamic human behaviour capturing how people change their behaviour based on the disease dynamics and NPIs.

As a case study, we used different health data from Ontario to evaluate our model predictions. However, our modelling framework can be easily adapted to any other country or province for which relevant data are available. Our modelling approach can provide important insights into how NPIs and vaccination can influence the health decisions people make during epidemics, and better understand how disease dynamics are affected by those decisions.

## Figures and Tables

**Figure 6.**
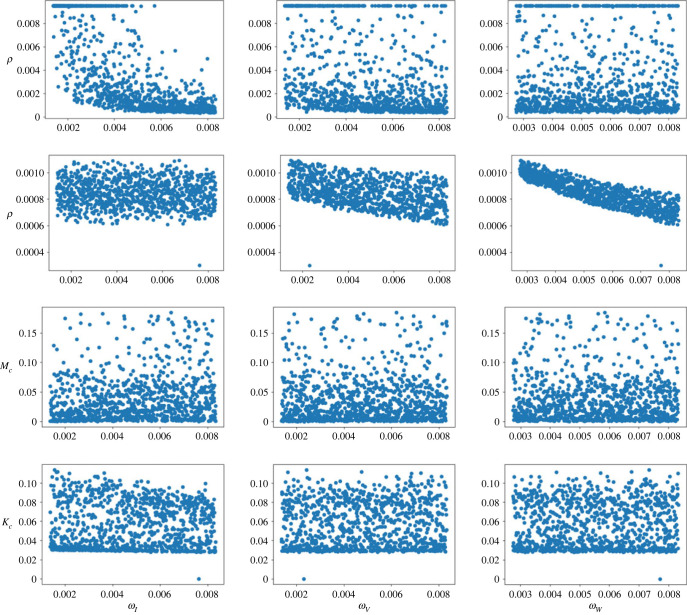
Scatterplots of the parameters *K*_*c*_, *M*_*c*_, *ρ*, *p* versus *ω*_*I*_, *ω*_*V*_ and *ω*_*W*_.

## Data Availability

All data are originally sourced at https://data.ontario.ca/en/dataset/status-of-covid-19-cases-in-ontario, https://data.ontario.ca/en/dataset/covid-19-vaccine-data-in-ontario and https://www.covid19immunitytaskforce.ca/seroprevalence-in-canada/. Data and relevant code for this research work are stored in GitHub: https://github.com/jetamolla3/PIs-and-NPIs-for-Controlling-the-COVID-19-Pandemic and have been archived within the Zenodo repository: http://doi.org/10.5281/zenodo.10200949 [48]. Supplementary material is available online [49].

## Appendix A. Differential equation models

We list the full differential equation model used in this analysis. The model is graphically depicted in figure 1. The disease progression starts with a susceptible group $S_{i}^{\xi }$ who, upon infection, transitions to an exposed group $E_{i}^{\xi }$. These people are not yet infectious and would not test positive, but are no longer susceptible. An exposed person transitions to a presymptomatic state, $P_{i}^{\xi }$ where they are infectious but do not present symptoms. This is the first state where someone could test positive and be isolated ($P_{M}^{\xi }$). A presymptomatic individual will transition to the full disease and continue to show no symptoms ($I_{A_{i}}^{\xi }$) or develop symptoms ($I_{S_{i}}^{\xi }$) and can be removed from testing ($I_{A_{M}}^{\xi }$ and $I_{S_{M}}^{\xi }$ respectively). Finally, the individual enters a recovery state where they are no longer infectious, but also not susceptible to the disease. This is $R_{A_{i}}^{\xi }$ ($R_{A_{M}}^{\xi }$ if isolated from testing) for those without symptoms and $R_{S_{i}}^{\xi }$ ($R_{S_{M}}^{\xi }$ if isolated from testing) for those with symptoms. The subscript in each disease states indicates a social distancing status with 0 being no social distancing (full contact probability), 1 being moderate social distancing (reduce contacts by a factor *δ*), and 2 being full social distancing (zero contact probability). The superscript in each disease state indicates the vaccination status with *u* indicating a person who has no vaccine protection and does not perceive themselves to have any protection, *v* indicating a person who has a successful vaccination and perceives themselves to have this immunity, and *w* indicating a person who does not have vaccine protection, but perceives an immunity. This can happen, for example, because they received a dose of vaccine, but it did not confer immunity.

The differential equation models for people in superscript state *u* (equation (A 1)), superscript state *v* (equation (A 2)), and superscript state *w* (equation (A 3)) are given by the following equations:

**A.1. Natural immunity, $X_{i}^{u}$**

A 1 $$\begin{array}{rl} & {\dot{S}}_{0}^{u}\;=-F_{S_{0}^{u}}-\mu S_{0}^{u}+\left(\frac{\nu }{2}\right)S_{1}^{u}+(1-q_{2})\nu S_{2}^{u}-pS_{0}^{u}+\omega_{W}S_{0}^{w}+\omega_{I}(R_{S_{0}^{u}}^{u}+R_{A_{0}^{u}}^{u}),\\ & {\dot{S}}_{1}^{u}=-F_{S_{1}^{u}}-\left(\frac{\mu }{2}\right)S_{1}^{u}+q_{1}\mu S_{0}^{u}-\left(\frac{\nu }{2}\right)S_{1}^{u}+q_{2}\nu S_{2}^{u}-pS_{1}+\omega_{W}S_{1}^{w}+\omega_{I}(R_{S_{1}^{u}}^{u}+R_{A_{1}^{u}}^{u}),\\ & {\dot{S}}_{2}^{u}=(1-q_{1})\mu S_{0}^{u}+\left(\frac{\mu }{2}\right)S_{1}^{u}-\nu S_{2}^{u}-pS_{2}^{u}+\omega_{W}S_{2}^{w}+\omega_{I}(R_{S_{2}^{u}}^{u}+R_{A_{2}^{u}}^{u}),\\ & {\dot{E}}_{0}^{u}=F_{S_{0}^{u}}-\mu E_{0}^{u}+\left(\frac{\nu }{2}\right)E_{1}^{u}+(1-q_{2})\nu E_{2}^{u}-\sigma E_{0}^{u}-pE_{0}^{u}+\omega_{W}E_{0}^{w},\\ & {\dot{E}}_{1}^{u}=F_{S_{1}^{u}}-\left(\frac{\mu }{2}\right)E_{1}^{u}+q_{1}\mu E_{0}^{u}-\left(\frac{\nu }{2}\right)E_{1}^{u}+q_{2}\nu E_{2}^{u}-\sigma E_{1}^{u}-pE_{1}^{u}+\omega_{W}E_{1}^{w},\\ & {\dot{E}}_{2}^{u}=(1-q_{1})\mu E_{0}^{u}+\left(\frac{\mu }{2}\right)E_{1}^{u}-\nu E_{2}^{u}-\sigma E_{2}^{u}-pE_{2}^{u}+\omega_{W}E_{2}^{w},\\ & {\dot{P}}_{0}^{u}=\sigma E_{0}^{u}-\mu P_{0}^{u}+\left(\frac{\nu }{2}\right)P_{1}^{u}+(1-q_{2})\nu P_{2}^{u}-\phi P_{0}^{u}-\rho_{a}P_{0}^{u}-pP_{0}^{u}+\omega_{W}P_{0}^{w},\\ & {\dot{P}}_{1}^{u}=\sigma E_{1}^{u}-\left(\frac{\mu }{2}\right)P_{1}^{u}+q_{1}\mu P_{0}^{u}-\left(\frac{\nu }{2}\right)P_{1}^{u}+q_{2}\nu P_{2}^{u}-\phi P_{1}^{u}-\rho_{a}P_{1}^{u}-pP_{1}^{u}+\omega_{W}P_{1}^{w},\\ & {\dot{P}}_{2}^{u}=\sigma E_{2}^{u}+(1-q_{1})\mu P_{0}^{u}+\left(\frac{\mu }{2}\right)P_{1}^{u}-\nu P_{2}^{u}-\phi P_{2}^{u}-\rho_{a}P_{2}^{u}-pP_{2}^{u}+\omega_{W}P_{2}^{w},\\ & {\dot{P}}_{M}^{u}=\rho_{a}(P_{0}^{u}+P_{1}^{u}+P_{2}^{u})-\phi P_{M}^{u}+\omega_{W}P_{M}^{w},\\ & {\dot{I}}_{S_{0}}^{u}=Q\phi P_{0}^{u}-\mu_{I}I_{S_{0}}^{u}-\gamma I_{S_{0}}^{u}-\rho_{s}I_{S_{0}}^{u}+\omega_{W}I_{S_{0}}^{w},\\ & {\dot{I}}_{S_{1}}^{u}=Q\phi P_{1}^{u}+q_{I}\mu_{I}I_{S_{0}}^{u}-\gamma I_{S_{1}}^{u}-\rho_{s}I_{S_{1}}^{u}+\omega_{W}I_{S_{1}}^{w},\\ & {\dot{I}}_{S_{2}}^{u}=Q\phi P_{2}^{u}+(1-q_{I})\mu_{I}I_{S_{0}}^{u}-\gamma I_{S_{2}}^{u}-\rho_{s}I_{S_{2}}^{u}+\omega_{W}I_{S_{2}}^{w},\\ & {\dot{I}}_{S_{M}^{u}}=\rho_{s}(I_{S_{0}}^{u}+I_{S_{1}}^{u}+I_{S_{2}}^{u})+Q\phi P_{M}^{u}-\gamma I_{S_{M}}^{u}+\omega_{W}I_{S_{M}}^{w},\\ & {\dot{I}}_{A_{0}^{u}}=(1-Q)\phi P_{0}^{u}-\mu I_{A_{0}}^{u}+\left(\frac{\nu }{2}\right)I_{A_{1}}^{u}+(1-q_{2})\nu_{2}I_{A_{2}}^{u}-\gamma I_{A_{0}}^{u}-\rho_{a}I_{A_{0}}^{u}-pI_{A_{0}}^{u}+\omega_{W}I_{A_{0}}^{w},\\ & {\dot{I}}_{A_{1}}^{u}=(1-Q)\phi P_{1}^{u}-\left(\frac{\mu }{2}\right)I_{A_{1}}^{u}+q_{1}\mu I_{A_{0}}^{u}-\left(\frac{\nu }{2}\right)I_{A_{1}}^{u}+q_{2}\nu I_{A_{2}}^{u}-\gamma I_{A_{1}}^{u}-\rho_{a}I_{A_{1}}^{u}-pI_{A_{1}}^{u}+\omega_{W}I_{A_{1}}^{w},\\ & {\dot{I}}_{A_{2}}^{u}=(1-Q)\phi P_{2}^{u}+(1-q_{1})\mu I_{A_{0}}^{u}+\left(\frac{\mu }{2}\right)I_{A_{1}}^{u}-\nu I_{A_{2}}^{u}-\gamma I_{A_{2}}^{u}-\rho_{a}I_{A_{2}}^{u}-pI_{A_{2}}^{u}+\omega_{W}I_{A_{2}}^{w},\\ & {\dot{I}}_{A_{M}}^{u}=\rho_{a}(I_{A_{0}}^{u}+I_{A_{1}}^{u}+I_{A_{2}}^{u})+(1-Q)\phi P_{M}^{u}\gamma I_{A_{M}}^{u}+\omega_{W}I_{A_{M}}^{w},\\ & {\dot{R}}_{S_{0}}^{u}=\gamma I_{S_{0}}^{u}-pR_{S_{0}}^{u}-\omega_{I}R_{S_{0}}^{u}+\omega_{W}R_{S_{0}}^{w}-\mu R_{S_{0}}^{u}+\left(\frac{\nu }{2}\right)R_{S_{1}}^{u}+\nu (1-q_{2})R_{S_{2}}^{u},\\ & {\dot{R}}_{S_{1}}^{u}=\gamma I_{S_{1}}^{u}-pR_{S_{1}}^{u}-\omega_{I}R_{S_{1}}^{u}+\omega_{W}R_{S_{1}}^{w}+\mu q_{1}R_{S_{0}}^{u}-\left(\frac{\mu }{2}\right)R_{S_{1}}^{u}-\left(\frac{\nu }{2}\right)R_{S_{1}}^{u}+\nu q_{2}R_{S_{2}}^{u},\\ & {\dot{R}}_{S_{2}}^{u}=\gamma I_{S_{2}}^{u}-pR_{S_{2}}^{u}-\omega_{I}R_{S_{2}}^{u}+\omega_{W}R_{S_{2}}^{w}+\left(\frac{\mu }{2}\right)R_{S_{1}}^{u}+\mu (1-q_{1})R_{S_{0}}^{u}-\nu R_{S_{2}}^{u},\\ & {\dot{R}}_{S_{M}^{u}}=\gamma I_{S_{M}}^{u}-pR_{S_{M}}^{u}-\omega_{I}R_{S_{M}}^{u}+\omega_{W}R_{S_{M}}^{w},\\ & {\dot{R}}_{A_{0}}^{u}=\gamma I_{A_{0}}-pR_{A_{0}}^{u}-\omega_{I}R_{A_{0}}^{u}+\omega_{W}R_{A_{0}}^{w}-\mu R_{A_{0}}^{u}+\left(\frac{\nu }{2}\right)R_{A_{1}}^{u}+\nu (1-q_{2})R_{A_{2}}^{u},\\ & {\dot{R}}_{A_{1}^{u}}=\gamma I_{A_{1}}^{u}-pR_{A_{1}}^{u}-\omega_{I}R_{A_{1}}^{u}+\omega_{W}R_{A_{1}}^{w}+\mu q_{1}R_{A_{0}}^{u}-\left(\frac{\mu }{2}\right)R_{A_{1}}^{u}-\left(\frac{\nu }{2}\right)R_{A_{1}}^{u}+\nu q_{2}R_{A_{2}}^{u},\\ & {\dot{R}}_{A_{2}}=\gamma I_{A_{2}}^{u}-pR_{A_{2}}^{u}-\omega_{I}R_{A_{2}}^{u}+\omega_{W}R_{A_{2}}^{w}+\left(\frac{\mu }{2}\right)R_{A_{1}}^{u}+\mu (1-q_{1})R_{A_{0}}^{u}-\nu R_{A_{2}}^{u}\\ \mathrm{and}\; & {\dot{R}}_{A_{M}}^{u}=\gamma I_{A_{M}}^{u}-pR_{A_{M}}^{u}-\omega_{I}R_{A_{M}}^{u}+\omega_{W}R_{A_{M}}^{w}. \end{array}\}$$

**A.2. Vaccine/perceived induced immunity, $X_{i}^{v}$**

A 2 $$\begin{array}{rl} & {\dot{S}}_{0}^{v}=-F_{S_{0}^{v}}-\mu S_{0}^{v}+\left(\frac{\nu }{2}\right)S_{1}^{v}+(1-q_{2})\nu S_{2}^{v}+q_{v}pS_{0}^{u}-\omega_{V}S_{0}^{v},\\ & {\dot{S}}_{1}^{v}=-F_{S_{1}^{v}}-\left(\frac{\mu }{2}\right)S_{1}^{v}+q_{1}\mu S_{0}^{v}-\left(\frac{\nu }{2}\right)S_{1}^{v}+q_{2}\nu S_{2}^{v}+q_{v}pS_{1}^{u}-\omega_{V}S_{1}^{v},\\ & {\dot{S}}_{2}^{v}=(1-q_{1})\mu S_{0}^{v}+\left(\frac{\mu }{2}\right)S_{1}^{v}-\nu S_{2}^{v}+q_{v}pS_{2}^{u}-\omega_{V}S_{2}^{v},\\ & {\dot{E}}_{0}^{v}=F_{V_{0}}-\mu E_{0}^{v}+\nu E_{1}^{v}+(1-q_{2})\nu E_{2}^{v}-\sigma E_{0}^{v}+q_{v}pE_{0}^{u},\\ & {\dot{E}}_{1}^{v}=F_{V_{1}}-\left(\frac{\mu }{2}\right)E_{1}^{v}+q_{1}\mu E_{0}^{v}-\left(\frac{\nu }{2}\right)E_{1}^{v}+q_{2}\nu E_{2}^{v}-\sigma E_{1}^{v}+q_{v}pE_{1}^{u},\\ & {\dot{E}}_{2}^{v}=(1-q_{1})\mu E_{0}^{v}+\left(\frac{\mu }{2}\right)E_{1}^{v}-\nu E_{2}^{v}-\sigma E_{2}^{v}+q_{v}pE_{2}^{u},\\ & {\dot{P}}_{0}^{v}=\sigma E_{0}^{v}-\mu P_{0}^{v}+\left(\frac{\nu }{2}\right)P_{1}^{v}+(1-q_{2})\nu P_{2}^{v}-\phi P_{0}^{v}-\rho_{a}^{v}P_{0}^{v}+q_{v}pP_{0}^{u},\\ & {\dot{P}}_{1}^{v}=\sigma E_{1}^{v}-\left(\frac{\mu }{2}\right)P_{1}^{v}+q_{1}\mu P_{0}^{v}-\left(\frac{\nu }{2}\right)P_{1}^{v}+q_{2}\nu P_{2}^{v}-\phi P_{1}^{v}-\rho_{a}^{v}P_{1}^{v}+q_{v}pP_{1}^{u},\\ & {\dot{P}}_{2}^{v}=\sigma E_{2}^{v}+(1-q_{1})\mu P_{0}^{v}+\left(\frac{\mu }{2}\right)P_{1}^{v}-\nu P_{2}^{v}-\phi P_{2}^{v}-\rho_{a}^{v}P_{2}^{v}+q_{v}pP_{2}^{u},\\ & {\dot{P}}_{M}^{v}=\rho_{a}^{v}(P_{0}^{v}+P_{1}^{v}+P_{2}^{v})-\phi P_{M}^{v},\\ & {\dot{I}}_{S_{0}}^{v}=Q\phi P_{0}^{v}-\mu_{I}I_{S_{0}}^{v}-\gamma I_{S_{0}}^{v}-\rho_{s}^{v}I_{S_{0}}^{v},\\ & {\dot{I}}_{S_{1}}^{v}=Q\phi P_{1}^{v}+q_{I}\mu_{I}I_{S_{0}}^{v}-\gamma I_{S_{1}}^{v}-\rho_{s}^{v}I_{S_{1}}^{v},\\ & {\dot{I}}_{S_{2}}^{v}=Q\phi P_{2}^{v}+(1-q_{I})\mu_{I}I_{S_{0}}^{v}-\gamma I_{S_{2}}^{v}-\rho_{s}^{v}I_{S_{2}}^{v},\\ & {\dot{I}}_{S_{M}}^{v}=\rho_{s}^{v}(I_{S_{0}}^{v}+I_{S_{1}}^{v}+I_{S_{2}}^{v})+Q^{v}\phi P_{M}^{v}-\gamma I_{S_{M}}^{v},\\ & {\dot{I}}_{A_{0}}^{v}=(1-Q)\phi P_{0}^{v}-\mu I_{A_{0}}^{v}+\left(\frac{\nu }{2}\right)I_{A_{1}}^{v}+(1-q_{2})\nu I_{A_{2}}^{v}-\gamma I_{A_{0}}^{v}-\rho_{a}^{v}I_{A_{0}}^{v}+q_{v}pI_{A_{0}}^{u},\\ & {\dot{I}}_{A_{1}}^{v}=(1-Q)\phi P_{1}^{v}-\left(\frac{\mu }{2}\right)I_{A_{1}}^{v}+q_{1}\mu I_{A_{0}}^{v}-\left(\frac{\nu }{2}\right)I_{A_{1}}^{v}+q_{2}\nu I_{A_{2}}^{v}-\gamma I_{A_{1}}^{v}-\rho_{a}^{v}I_{A_{1}}^{v}+q_{v}pI_{A_{1}}^{u},\\ & {\dot{I}}_{A_{2}}^{v}=(1-Q)\phi P_{2}^{v}+(1-q_{1})\mu I_{A_{0}}^{v}+\left(\frac{\mu }{2}\right)I_{A_{1}}^{v}-\nu I_{A_{2}}^{v}-\gamma I_{A_{2}}^{v}-\rho_{a}^{v}I_{A_{2}}^{v}+q_{v}pI_{A_{2}}^{u},\\ & {\dot{I}}_{A_{M}}^{v}=\rho_{a}^{v}(I_{A_{0}}^{v}+I_{A_{1}}^{v}+I_{A_{2}}^{v})+(1-q_{v})\phi P_{M}^{v}-\gamma I_{A_{M}}^{v},\\ & {\dot{R}}_{S_{0}}^{v}=\gamma I_{S_{0}}^{v}+q_{v}pR_{S_{0}}^{u}-\omega_{I}R_{S_{0}}^{v}-\mu R_{S_{0}}^{v}+\left(\frac{\nu }{2}\right)R_{S_{1}}^{v}+\nu (1-q_{2})R_{S_{2}}^{v},\\ & {\dot{R}}_{S_{1}}^{v}=\gamma I_{S_{1}}^{v}+q_{v}pR_{S_{1}}^{u}-\omega_{I}R_{S_{1}}^{v}+\mu q_{1}R_{S_{0}}^{v}-\left(\frac{\mu }{2}\right)R_{S_{1}}^{v}-\left(\frac{\nu }{2}\right)R_{S_{1}}^{v}+\nu q_{2}R_{S_{2}}^{v},\\ & {\dot{R}}_{S_{2}}^{v}=\gamma I_{S_{2}}^{v}+q_{v}pR_{S_{2}}^{u}-\omega_{I}R_{S_{2}}^{v}+\left(\frac{\mu }{2}\right)R_{S_{1}}^{v}+\mu (1-Q)R_{S_{0}}^{v}-\nu_{2}R_{S_{2}}^{v},\\ & {\dot{R}}_{S_{M}}^{v}=\gamma I_{S_{M}}^{v}+q_{v}pR_{S_{M}}^{u}-\omega_{I}R_{S_{M}}^{v},\\ & {\dot{R}}_{A_{0}}^{v}=\gamma I_{A_{0}}^{v}+q_{v}pR_{A_{0}}^{u}-\omega_{I}R_{A_{0}}^{v}-\mu R_{A_{0}}^{v}+\left(\frac{\nu }{2}\right)R_{A_{1}}^{v}+\nu_{2}(1-q_{2})R_{A_{2}}^{v},\\ & {\dot{R}}_{A_{1}}^{v}=\gamma I_{A_{1}}^{v}+q_{v}pR_{A_{1}}^{u}-\omega_{I}R_{A_{1}}^{v}+\mu q_{1}R_{A_{0}}^{v}-\left(\frac{\mu }{2}\right)R_{A_{1}}^{v}-\left(\frac{\nu }{2}\right)R_{A_{1}}^{v}+\nu q_{2}R_{A_{2}}^{v},\\ & {\dot{R}}_{A_{2}}^{v}=\gamma I_{A_{2},}^{v}+q_{v}pR_{A_{2}}^{u}-\omega_{I}R_{A_{2}}^{v}+\left(\frac{\mu }{2}\right)R_{A_{1}}^{v}+\mu (1-Q)R_{A_{0}}^{v}-\nu R_{A_{2}}^{v},\\ \mathrm{and}\;\; & {\dot{R}}_{A_{M}}^{v}=\gamma I_{A_{M}}^{v}+q_{v}pR_{A_{M}}^{u}-\omega_{I}R_{A_{M}}^{v}. \end{array}\}$$

**A.3. Perceived induced immunity, $X_{i}^{w}$**

A 3 $$\begin{array}{rl} & {\dot{S}}_{0}^{w}=-F_{S_{0}^{w}}-\mu S_{0}^{w}+\left(\frac{\nu }{2}\right)S_{1}^{w}+(1-q_{2})\nu S_{2}^{w}+(1-q_{v})pS_{0}^{u}+\omega_{I}(R_{S_{0}}^{v}+R_{S_{0}}^{w}+R_{A_{0}}^{v}+R_{A_{0}}^{w}+R_{A_{M}}^{v}\\ & \;+R_{A_{M}}^{w}+R_{S_{M}}^{v}+R_{S_{M}}^{w}+R_{S_{M}}^{u}+R_{A_{M}}^{u})-\omega_{W}S_{0}^{w}+\omega_{V}S_{0}^{v},\\ & {\dot{S}}_{1}^{w}=-F_{S_{1}^{w}}-\left(\frac{\mu }{2}\right)S_{1}^{w}+q_{1}\mu S_{0}^{w}-\left(\frac{\nu }{2}\right)S_{1}^{w}+q_{2}\nu S_{2}^{w}+(1-q_{v})pS_{1}^{u}\\ & \;+\omega_{I}(R_{S_{1}}^{v}+R_{S_{1}}^{w}+R_{A_{1}}^{v}+R_{A_{1}}^{w})-\omega_{W}S_{1}^{w}+\omega_{V}S_{1}^{v},\\ & {\dot{S}}_{2}^{w}=(1-q_{1})\mu S_{0}^{w}+\left(\frac{\mu }{2}\right)S_{1}^{w}-\nu S_{2}^{w}+(1-q_{v})pS_{2}^{w}+\omega_{I}(R_{S_{2}}^{v}+R_{S_{2}}^{w}+R_{A_{2}}^{v}+R_{A_{2}}^{w})-\omega_{W}S_{2}^{w}+\omega_{V}S_{2}^{v},\\ & {\dot{E}}_{0}^{w}=F_{W_{0}}-\mu E_{0}^{w}+\left(\frac{\nu }{2}\right)E_{1}^{w}+(1-q_{2})\nu E_{2}^{w}-\sigma E_{0}^{w}+(1-q_{v})pE_{0}-\omega_{W}E_{0}^{w},\\ & {\dot{E}}_{1}^{w}=F_{W_{1}}-\left(\frac{\mu }{2}\right)E_{1}^{w}+q_{1}\mu E_{0}^{w}-\left(\frac{\nu }{2}\right)E_{1}^{w}+q_{2}\nu E_{2}^{w}-\sigma E_{1}^{w}+(1-q_{v})pE_{1}-\omega_{W}E_{1}^{w},\\ & {\dot{E}}_{2}^{w}=(1-q_{1})\mu E_{0}^{w}+\left(\frac{\mu }{2}\right)E_{1}^{w}-\nu E_{2}^{w}-\sigma E_{2}^{w}+(1-q_{v})pE_{2}-\omega_{W}E_{2}^{w},\\ & {\dot{P}}_{0}^{w}=\sigma E_{0}^{w}-\mu P_{0}^{w}+\left(\frac{\nu }{2}\right)P_{1}^{w}+(1-q_{2})\nu P_{2}^{w}-\phi P_{0}^{w}-\rho_{a}^{v}P_{0}^{w}+(1-q_{v})pP_{0}-\omega_{W}P_{0}^{w},\\ & {\dot{P}}_{1}^{w}=\sigma E_{1}^{w}-\left(\frac{\mu }{2}\right)P_{1}^{w}+q_{1}\mu P_{0}^{w}-\left(\frac{\nu }{2}\right)P_{1}^{w}+q_{2}\nu P_{2}^{w}-\phi P_{1}^{w}-\rho_{a}^{v}P_{1}^{w}+(1-q_{v})pP_{1}-\omega_{W}P_{1}^{w},\\ & {\dot{P}}_{2}^{w}=\sigma E_{2}^{w}+(1-q_{1})\mu P_{0}^{w}+\left(\frac{\mu }{2}\right)P_{1}^{w}-\nu P_{2}^{w}-\phi P_{2}^{w}-\rho_{a}^{v}P_{2}^{w}+(1-q_{v})pP_{2}-\omega_{W}P_{2}^{w},\\ & {\dot{P}}_{M}^{w}=\rho_{a}^{v}(P_{0}^{w}+P_{1}^{w}+P_{2}^{w})-\phi P_{M}^{w}-\omega_{W}P_{M}^{w},\\ & {\dot{I}}_{S_{0}}^{w}=Q\phi P_{0}^{w}-\mu_{I}I_{S_{0}}^{w}-\gamma I_{S_{0}}^{w}-\rho_{s}^{v}I_{S_{0}}^{w}-\omega_{W}I_{S_{0}}^{w},\\ & {\dot{I}}_{S_{1}}^{w}=Q\phi P_{1}^{w}+q_{I}\mu_{I}I_{S_{0}}^{w}-\gamma I_{S_{1}}^{w}-\rho_{s}^{v}I_{S_{1}}^{w}-\omega_{W}I_{S_{1}}^{w},\\ & {\dot{I}}_{S_{2}}^{w}=Q\phi P_{2}^{w}+(1-q_{I})\mu_{I}I_{S_{0}}^{w}-\gamma I_{S_{2}}^{w}-\rho_{s}^{v}I_{S_{2}}^{w}-\omega_{W}I_{S_{2}}^{w},\\ & {\dot{I}}_{S_{M}}^{w}=\rho_{s}^{v}(I_{S_{0}}^{w}+I_{S_{1}}^{w}+I_{S_{2}}^{w})+Q\phi P_{M}^{w}-\gamma I_{S_{M}}^{w}-\omega_{W}I_{S_{M}}^{w},\\ & {\dot{I}}_{A_{0}}^{w}=(1-Q)\phi P_{0}^{w}-\mu I_{A_{0}}^{w}+\left(\frac{\nu }{2}\right)I_{A_{1}}^{w}+(1-q_{2})\nu I_{A_{2}}^{w}-\gamma I_{A_{0}}^{w}-\rho_{a}^{v}I_{A_{0}}^{w}+(1-q_{v})pI_{A_{0}}-\omega_{W}I_{A_{0}}^{w},\\ & {\dot{I}}_{A_{1}}^{w}=(1-Q)\phi P_{1}^{w}-\mu I_{A_{1}}^{w}+q_{1}\mu I_{A_{0}}^{w}-\left(\frac{\nu }{2}\right)I_{A_{1}}^{w}+q_{2}\nu I_{A_{2}}^{w}-\gamma I_{A_{1}}^{w}-\rho_{a}^{v}I_{A_{1}}^{w}+(1-q_{v})pI_{A_{1}}-\omega_{W}I_{A_{1}}^{w},\\ & {\dot{I}}_{A_{2}}^{w}=(1-Q)\phi P_{2}^{w}+(1-q_{1})\mu I_{A_{0}}^{w}+\left(\frac{\mu }{2}\right)I_{A_{1}}^{w}-\nu_{2}I_{A_{2}}^{w}-\gamma I_{A_{2}}^{w}-\rho_{a}^{v}I_{A_{2}}^{w}+(1-q_{v})pI_{A_{2}}-\omega_{W}I_{A_{2}}^{w},\\ & {\dot{I}}_{A_{M}}^{w}=\rho_{a}^{v}(I_{A_{0}}^{w}+I_{A_{1}}^{w}+I_{A_{2}}^{w})+(1-Q)\phi P_{M}^{w}-\gamma I_{A_{M}}^{w}-\omega_{W}I_{A_{M}}^{w},\\ & {\dot{R}}_{S_{0}}^{w}=\gamma I_{S_{0}}^{w}+(1-q_{v})pR_{S_{0}}^{u}-\omega_{I}R_{S_{0}}^{w}-\omega_{W}R_{S_{0}}^{w}-\mu R_{S_{0}}^{w}+\left(\frac{\nu }{2}\right)R_{S_{1}}^{w}+\nu (1-q_{2})R_{S_{2}}^{w},\\ & {\dot{R}}_{S_{1}}^{w}=\gamma I_{S_{1}}^{w}+(1-q_{v})pR_{S_{1}}^{u}-\omega_{I}R_{S_{1}}^{w}-\omega_{W}R_{S_{1}}^{w}+\mu q_{1}R_{S_{0}}^{w}-\left(\frac{\mu }{2}\right)R_{S_{1}}^{w}-\left(\frac{\nu }{2}\right)R_{S_{1}}^{w}+\nu q_{2}R_{S_{2}}^{w},\\ & {\dot{R}}_{S_{2}}^{w}=\gamma I_{S_{2}}^{w}+(1-q_{v})pR_{S_{2}}^{u}-\omega_{I}R_{S_{2}}^{w}-\omega_{W}R_{S_{2}}^{w}+\left(\frac{\mu }{2}\right)R_{S_{1}}^{w}+\mu (1-q_{1})R_{S_{0}}^{w}-\nu R_{2}^{w},\\ & {\dot{R}}_{S_{M}}^{w}=\gamma I_{S_{M}}^{w}+(1-q_{v})pR_{S_{M}}^{u}-\omega_{I}R_{S_{M}}^{w}-\omega_{W}R_{S_{M}}^{w},\\ & {\dot{R}}_{A_{0}}^{w}=\gamma I_{A_{0}}^{w}+(1-q_{v})pR_{A_{0}}^{u}-\omega_{I}R_{A_{0}}^{w}-\omega_{W}R_{A_{0}}^{w}-\mu R_{A_{0}}^{w}+\left(\frac{\nu }{2}\right)R_{A_{1}}^{w}+\nu (1-q_{2})R_{A_{2}}^{w},\\ & {\dot{R}}_{A_{1}}^{w}=\gamma I_{A_{1}}^{w}+(1-q_{v})pR_{A_{1}}^{u}-\omega_{I}R_{A_{1}}^{w}-\omega_{W}R_{A_{1}}^{w}+\mu q_{1}R_{A_{0}}^{w}-\left(\frac{\mu }{2}\right)R_{A_{1}}^{w}-\left(\frac{\nu }{2}\right)R_{A_{1}}^{w}+\nu q_{2}R_{A_{2}}^{w},\\ & {\dot{R}}_{A_{2}}^{w}=\gamma I_{A_{2},}^{w}+(1-q_{v})pR_{A_{2}}^{u}-\omega_{I}R_{A_{2}}^{w}-\omega_{W}R_{A_{2}}^{w}+\left(\frac{\mu }{2}\right)R_{A_{1}}^{w}+\mu (1-Q)R_{A_{0}}^{w}-\nu R_{A_{2}}^{w},\\ \mathrm{and}\; & {\dot{R}}_{A_{M}}^{w}=\gamma I_{A_{M},}^{w}+(1-q_{v})pR_{A_{M}}^{u}-\omega_{I}R_{A_{M}}^{w}-\omega_{W}R_{A_{M}}^{w} \end{array}\}$$

where $\left\{F_{S_{0}^{u}},F_{S_{1}^{u}},F_{S_{0}^{v}},F_{S_{1}^{v}},F_{S_{0}^{w}},F_{S_{1}^{w}}\right\}$ are force of infections defined by

$$F_{\xi_{0}}=\frac{N_{\mathrm{crit}}\beta \xi_{0}}{N}F;\;\xi_{0}=S_{0}^{u},S_{0}^{v},S_{0}^{w}$$

and

$$F_{\xi_{1}}=\frac{N_{\mathrm{crit}}\delta \beta \xi_{1}}{N}F;\;\xi_{1}=S_{1}^{u},S_{1}^{v},S_{1}^{w},$$

with

$$\begin{array}{rl} & F=(I_{S_{0}}^{u}+I_{S_{0}}^{v}+I_{S_{0}}^{w})+\alpha (P_{0}^{u}+I_{A_{0}}^{u}+P_{0}^{v}+I_{A_{0}}^{v}+P_{0}^{w}+I_{A_{0}}^{w}+\delta (P_{1}^{u}+I_{A_{1}}^{u}+P_{1}^{v}+I_{A_{1}}^{v}+P_{1}^{w}+I_{A_{1}}^{w}))\\ & \;+\delta (I_{S_{1}}^{u}+I_{S_{1}}^{v}+I_{S_{1}}^{w}). \end{array}$$

All parameters used in the model are provided in table 2 of the main text.

## Appendix B. Sensitivity analysis

In figure 6, we present the scatterplots for the estimated parameters *K*_*c*_, *M*_*c*_, *ρ* and *p* versus the waning rates *ω*_*I*_, *ω*_*V*_ and *ω*_*W*_. The results show that there is no relationship between *K*_*c*_, *M*_*c*_, *ρ* and *ω*_*I*_, *ω*_*V*_, *ω*_*W*_. The waning rates *ω*_*I*_, *ω*_*V*_ and *ω*_*W*_ determine how fast the infection or vaccine-induced immunity, and the perceived-induced immunity wane, and it would be expected that they do not influence the values of the behaviour parameters *ρ*, *M*_*c*_, *K*_*c*_ since individuals do not know when their immunity wanes. While there is no relationship between *p* and *ω*_*I*_, the results indicate a negative relationship of moderate strength between *p* and *ω*_*V*_, and *p* and *ω*_*W*_. This implies that as the rate at which individuals transition from *u* to *w* decreases, the vaccination rate has to increase to fit the vaccination data.
